# Nursing labor supply in Iran: a survey in Shiraz public hospitals in 2022

**DOI:** 10.1186/s12962-024-00542-3

**Published:** 2024-04-22

**Authors:** Ali Keshavarzi, Sajad Delavari, Farhad Lotfi, Zahra Goudarzi, Faezeh Bashiri, Mohsen Bayati

**Affiliations:** 1 Student Research Committee, School of Health Management and Information Sciences, Shiraz University of Medical Sciences, Shiraz, Iran; 2 Health Human Resources Research Center, School of Health Management and Information Sciences, Shiraz University of Medical Sciences, Almas Building, Alley 29, Qasrodasht Ave, Shiraz, 71336-54361 Iran

**Keywords:** Labor supply, Health manpower, Nursing, Shortage, Factor determinants

## Abstract

**Background:**

The labor supply of nurses, as one of the main healthcare workers, is an important issue in health human resources planning in all health systems. Finding the factors affecting it, could help policymakers to solve the shortage of nursing work supply. The present study aimed to investigating the quantity and factors affecting the nurses' labor supply in Iran.

**Method:**

In this cross-sectional study, a sample of 598 nurses working in public hospitals of Shiraz (Iran) were selected via proportionate stratified random sampling method. The required data was collected using a structured questionnaire which asked working hours and other related factors. To analyze the data, descriptive statistics, univariate analysis and multivariate linear regression were performed using STATA 15. The multivariate labor supply model was estimated separately for married and single nurses.

**Results:**

The average weekly working hours of nurses was 54.65 h in all medical centers and 50.28 h in the main hospital. The regression results showed that the labor supply of nurses with work experience (β = − 0.368, P = 0.014), satisfaction with work shift arrangement (β = − 2.473, P = 0.001), income between 60-89 million rial (β = − 14.046, P = 0.002), income between  90-119 million rial(β = − 12.073, P = 0.012), and working in the emergency department (β = − 5.043, P = 0.017) had negative and significant relationship; But there was a positive and significant relationship with satisfaction of the work environment (β = 1.86, P = 0.011), workload at work (β = 1.951, P = 0.023) and employment status (contractual employees) (β = 4.704, P = 0.004).

**Conclusion:**

The labor supply function of nurses is affected by demographic, economic and non-economic factors. The most contributing factors were related to non-economic variables. It seems that the non-financial cost and benefits related to the job as well as internal factors have more important role on the nurses' labor supply.

## Introduction

Human resources are one of the most valuable resources of a hospital and any other organization [1]. Nurses constitute a major part of the workforce working in the healthcare system, who play an important role in providing direct care to the patient. Having a sufficient number of nurses is one of the necessities of the health system in any society [2]. Therefore, due to the professionalism of their working, they should be given multi-dimensional and comprehensive attention in order to get the necessary benefit from their services. In addition, due to the significant costs of training nursing staff, their efficiency and willingness to provide service and supply of work is particularly important from an economic point of view [3].

Labor supply represents the population of working age people who are willing and able to work. Labor supply in the micro field shows the number of available person-hours in market, and in the macro level, it means the flow of labor reserves [4]. The low supply of work by nurses creates many problems for health systems and makes them face the challenge of a shortage of nurses [3]. The World Health Organization (WHO) in a report about the challenge of nurse shortage (2010) stated that although the issue of nurse shortage has similar consequences in all countries, the consequences are more serious in developing countries. Because in developed countries, this problem is compensated by recruiting from poorer countries, but in developing countries, this is not possible due to lack of financial ability and economic weakness [5]. One of the consequences of the nurse’s shortage is the impact on delivery and quality of health services [6]. In the United Kingdom (UK), the lack of nurses led to increased waiting times for surgeries and delays in essential care, and even the closure of some departments [7].

Various factors can affect the labor supply of nurses. Antonazo [8] showed that the wage has a positive effect, but the household’s non-work income, spouse wage, the number of children, especially children under 5 years old, age and education also have a negative effect on the labor supply of nurses. Condliffeet al.’s [9] in the United States (US) showed that the increase in family income is associated with a decrease in the work of nurses, and emphasized the negative effect of age and the presence of a young child on the willingness of nurses to work. Borjas [10] considered the housework and the employment status of spouses to be effective on the labor supply of nurses. Kankanranta and Rissanen [11], states that other than wage, non-financial factors can also motive nurses’ to increase working hours.

The problem of the nurse’s shortage in Iran is one of the challenges that health policymakers have always faced [12].According to a study in Iran in 2015, there is a shortage of 130,000 nurses, and it is predicted to have 200,000 nurses by 2020 [13]. The number of nurses per hospital bed in different provinces of Iran is 0.6 (less than one nurse) to 1.5 (more than one nurse). Iran has a ratio of 1.5 nurses per thousand population. Iran has lower rank than Egypt, Turkey, Oman, Kuwait and Qatar [14]. The maximum essential motives for the mission of scarcity of nurses in Iran are negative social status, early retirement, immigration, willingness to desert the present day job, employment in different jobs, work-associated injuries, low employment rates and growth of health center beds [15]. Solving this problem, needs having a comprehensive look at the factors related to the supply and demand for nursing. Training more nursing staff will not necessarily lead to solving the shortage problem; rather, it is necessary to consider the factors that affect their desire to supply their work [16]. Identifying the factors affecting the labor supply by nurses and trying to remove the obstacles to increase the supply can help to solve the shortage in the supply of nursing work. Due to the importance of this issue, especially in developing countries, as well as the lack of a comprehensive study in this field in Iran, the present study was conducted with the aim of estimating the nurses’ labor supply and the factors affecting it in Shiraz.

Labor supply can be investigated according to two approaches, intensive and extensive. In this study, we used the perspective of intensive approach to investigate nursing labor supply. In other words, we studied the working hours (and factors affecting it) of nurses who are currently in the nurses labor market.

## Methods

In the current cross-sectional study, the study population included all nurses (in all nursing positions) working in public hospitals of Shiraz, a city with 1,955,500 population in southern part of Iran. The sample consisted of 598 nurses, who were selected by proportionate stratified sampling method in 14 public hospitals in Shiraz city. Thus, first, the number of nurses working in the public hospitals was determined, and according to the total sample size, the required number of samples from each hospital was determined. The samples selected in hospitals A to N were 33 (5.52%), 19 (3.18%), 34 (5.69%), 40 (6.69%), 81 (13.55%), 21 (3.51%), 26 (4.35%), 49 (8.19%), 61 (10.2%), 26 (4.35%), 19 (3.18%), 96 (16.05%), 48 (8.03%), and 45 (7.53%), respectively. Then, hospitals were visited in different work shifts and the questionnaire was given to the nurses, completed by them and finally collected. All distributed questionnaires were completed by selected nurses. It should be mentioned that data collection was done in the first six months of 2022.

The data collection tool was a researcher made questionnaire. For this purpose, a primary questionnaire was prepared based on theoretical foundations and existing literature in the field of factors affecting the labor supply of nurses [8, 9, 17].

The questionnaire had 32 questions including demographic factors (age, gender, education level, type of employment, marital status, number of children and the number of children under five years old), economic information (individual's income, spouse’s income and income from other sources), non-economic factors (physical environment, working conditions and work pressure, job satisfaction, work experience, sufficient number of nursing personnel working in the department, number of weekly working hours, volume and pressure of household affairs). The questions in most dimensions are closed-ended designed, but for some question about the weekly income of nurses, age, work experience, number of children, working department which were designed in an open-ended. Also, there was a question related to the patients' self-assessment of their economic status, which was designed as a Likert scale of 0 to 10, and the nurses had to determine the score based on that.

The validity of the questionnaire was checked and revised by a number of relevant experts. The reliability of the questionnaire was investigated using a pilot study. For this purpose, the questionnaire was given to 30 randomly selected nurses to be completed by them, and the reliability of the questionnaire was confirmed by using Cronbach’s alpha coefficient with a coefficient above 0.7.

Descriptive statistics, univariate analysis and multivariate linear regression were used to analyze the data. Factors that were significant in univariate analysis were included in the regression model. Factors affecting the labor supply of nurses that are included in the model as explanatory variables include three categories of demographic and social factors, economic factors and non-economic factors. The dependent variable of the model was the number of weekly working hours of nurses, which indicates the supply of nursing labor. Regression analysis was conducted separately by the married and single status of the nurses.

Analyzes were performed using STATA 15.

The study was found to be in accordance to the ethical principles and the national norms and standards for conducting medical research. The research protocol was approved by the Ethics Committee of Shiraz University of Medical Sciences under code IR.SUMS.NUMIMG.REC.1400.027.

## Results

The average weekly working hours of nurses was 54.65 h in all medical centers and 50.28 h in the main hospital. The minimum weekly working hours were 25 h and the maximum was 107 h. The mean work experience was 8.04 years and the average age of the nurses was 32.54 years. The number of family members was 2.43, in average. The mean number of children and children under five years old were 0.648 and 0.175, respectively. The average score of nurses on their economic situation from zero to 10 was equal to 3.41, which showed their dissatisfaction with their economic situation (Table 1).

**Table 1:** Descriptive statistics of quantitative study variables among nurses working in public hospitals in Shiraz in 2022

	Mean	Standard deviation	Min	Max
Weekly working hours in the main hospital	50.28	10.69	25	96
Weekly working hours in all medical centers	54.65	16.16	25	107
Number of children under five years old	0.175	0.412	0	3
Number of children	0.648	0.899	0	4
Household size	2.43	1.58	0	8
Work experience	8.04	6.42	0.4	28
Age	32.54	6.81	23	57
Economic status	3.41	1.83	0	10

Number of children, household size

Most of the participants were female (82.52%), married (56.42%), living in Shiraz (96.79%) with a medium and poor employment status (61.19%), non-household head (82.24%), having a bachelor’s degree (89.54%), income below 90 million Iranian Rials monthly (80.2%). Only 15.08% of nurses had a second job and 84.92% were only engaged in nursing. The majority of nurses were working only in one hospital. Among the participants, 3.26% were working in nursing management positions, 87.48% were working as nurse, and 9.26% were working as nursing assistants and other nursing positions. Also, according to most of the participants (78.22%), the number of ward nurses was insufficient in most hospitals. Nurses’ satisfaction with the arrangement of work shifts (39.6%) and the atmosphere of the work environment (40.24%) was average. About 41.68% of the nurses rated the volume and pressure of their workplace as too high and 36.27% of them rated the volume and pressure of household affairs as high. It should be noted that the number of nurses working in the general, internal, and surgical departments (38.63%) was more than other departments (Table 2).

**Table 2:** Descriptive statistics of categorical variables of the study among nurses working in public hospitals in Shiraz in 2022

Variable		Frequency	Percent	Variable		Frequency	Percent
Gender	Male	104	17.48	Working department	General, internal, and surgical	231	38.63
	Female	491	82.52		Pediatrics	45	7.53
Marital status	Single	258	43.58		ICU and CCU	183	30.60
	Married	334	56.42		Gynecology and maternity	29	4.58
Location	Shiraz	542	96.79		Emergency	83	13.88
	Other cities	18	3.21		Operation room	17	2.84
Employment status	Strong (official employees)	222	38.81		Administrative	10	1.67
	Medium (contractual employees)	302	52.52	Sufficiency of nursing personnel in department	Yes	127	21.78
	Weak (corporate employees)	48	8.39		No	456	78.22
Household head	Yes	92	17.76	Satisfaction with the arrangement of work shifts	Very high	21	3.52
	No	426	82.24		High	67	11.24
Education level	Diploma and under diploma	20	3.37		Moderate	236	39.60
	Post-diploma	6	1.01		Low	139	23.32
	Bachelor’s degree	531	89.54		Very low	133	22.32
	Master’s degree	35	5.90	Satisfaction with atmosphere of the work environment	Very high	28	4.71
	PhD	1	0.17		High	139	23.40
Income from nursing^a^	Below 59 Million Rial	36	6.04		Moderate	239	40.24
	Between 60 to 89 Million Rial	442	74.16		Low	112	18.86
	Between 90 to 119 Million Rial	102	17.11		Very low	76	12.79
	More than 120 Million Rial	16	2.68	Volume and pressure of household affairs	Very high	157	26.34
Spouse's income	No	294	50.87		High	219	36.74
	Yes	284	49.13		Moderate	165	27.68
Income from other sources	No	470	80.07		Low	45	7.55
	Yes	117	19.93		Very low	10	1.68
Job position	Manager such as supervisor	19	3.26	Workload of the workplace	Very high	248	41.68
	Nurse	510	87.48		High	243	40.84
	Nursing assistants	34	5.83		Moderate	77	12.94
	Other positions	20	3.43		Low	19	3.19
					Very low	8	1.34

^a^It should be noted that the exchange rate of Iranian Rial to US Dollar was about 0.000024 Rial based on the government exchange rate

Table 3 shows the regression results of nurses’ labor supply function for married nurses. Nurses’ labor supply had a negative and significant relationship with work experience, income groups between  60-119  million Rials, satisfaction with work shift arrangement. Labor supply had a positive and significant relationship with the nurse’s satisfaction with the work environment (at the ten percent level), work volume and pressure at the workplace and the greater the workload of the workplace, the more hours nurses had to work, which is a sign of a shortage of nurses. People with medium employment status (such as contractual employees) had significantly more and people with weak employment status (such as corporate employees) had insignificantly less labor supply than nurses with strong employment relationship (such as official employees). In terms of the place of work, nurses in the emergency department had significantly less labor supply. Table 4 shows the regression results of nurses’ labor supply function for single nurses. All variables did not show a significant relationship with nurses' labor supply.

**Table 3:** Regression estimates of factors affecting the labor supply of married nurses, Shiraz, 2022

Explanatory variables		Coefficient	P-value
Work experience		− 0.368	0.014
Gender (female)		1.531	0.107
Household size		0.786	0.172
Number of children		− 1.404	0.191
Children under five years old (No)		− 0.345	0.815
Sufficiency of nursing personnel number working in the department		− 0.738	0.658
Satisfaction with the arrangement of work shifts		− 2.473	0.001
Satisfaction with atmosphere of the work environment		1.860	0.011
Workload of the workplace		1.951	0.023
Volume and pressure of household affairs		− 0.7919	0.359
Employment type	Strong (official employees)	Reference	
	Medium (contractual employees)	4.704	0.004
	Weak (corporate employees)	− 3.577	0.129
Education level	Diploma and under diploma	Reference	
	Post-diploma	2.117	0.831
	Bachelor’s degree	10.252	0.351
	Master’s degree	0.285	0.979
	PhD	2.348	0.834
Job position	Manager such as supervisor	Reference	
	Nurse	− 2.094	0.610
	Nursing assistants	− 3.821	0.561
	Other positions	− 7.857	0.132
Income from nursing	Below 59 Million Rials	Reference	
	Between 60 to 89 Million Rials	− 14.046	0.002
	Between 90 to 119 Million Rials	− 12.073	0.012
	More than 120 Million Rials	− 7.860	0.193
Income from other sources		0.576	0.37
Spouse’s income		− 3.194	− 1.32
Economic status		− 0.485	− 1.22
Working department	General, internal, and surgical	Reference	
	Pediatrics	− 1.062	0.669
	ICU and CCU	− 1.044	0.501
	Gynecology and maternity	− 2.043	0.502
	Emergency	− 5.043	0.017
	Operation room	− 2.100	0.552
	Administrative	− 5.790	0.331

**Table 4:** Regression estimates of factors affecting the labor supply of single nurses, Shiraz, 2022

Explanatory variables		Coefficient	P-value
Work experience		− 0.0443	0.805
Gender (female)		1.761	0.375
Sufficiency of nursing personnel number working in the department		1.210	0.516
Satisfaction with the arrangement of work shifts		− 1.454	0.097
Satisfaction with atmosphere of the work environment		0.066	0.932
Workload of the workplace		0.571	0.522
Volume and pressure of household affairs		− 0.420	0.595
Employment type	Strong (official employees)	Reference	
	Medium (contractual employees)	1.351	0.437
	Weak (corporate employees)	− 6.065	0.052
Education level	Diploma and under diploma	Reference	
	Post-diploma	Omitted	Omitted
	Bachelor's degree	− 0.334	0.97
	Master's degree	5.031	.06
	PhD	Omitted	Omitted
Job position	Manager such as supervisor	Reference	
	Nurse	4.873	0.453
	Nursing assistants	16.841	0.074
	Other positions	3.095	0.703
Income from nursing	Below 59 Million Rials	Reference	
	Between 60 to 89 Million Rials	4.734	0.203
	Between 90 to 119 Million Rials	0.276	0.951
	More than 120 Million Rials	− 3.918	0.625
Income from other sources		0.345	0.864
Economic status		0.672	0.111
Working department	General, internal, and surgical	Reference	
	Pediatrics	− 2.596	0.342
	ICU and CCU	0.630	0.709
	Gynecology and maternity	− 5.370	0.210
	Emergency	− 0.459	0.829
	Operation room	− 4.727	0.483
	Administrative	9.039	0.461

## Discussion

Nursing is a vital profession in providing health care. In order to be able to attract new individuals to the nursing profession and also to prevent the departure of trained nurses and motivate them to provide more working hours, the basic challenges of the supply side in the labor market must be solved. Therefore, it is important to identify the determinants of nurses’ labor supply [11]. The purpose of this study was to investigate the factors affecting the labor supply of nursing workforce in Shiraz in 2022.

According to the results, most of the nurses had income between 70 and 90 million Rials per month, while the minimum salary in Iran was 53,082,820 in 2022.The findings of the present study showed that the nurses participating in this study had an average weekly working hour of 54.65 h. Mandatory working hours for nurses in Iran is 44 h per week according to the service instructions of the nurse’s group, and they can work overtime in the hospital up to 80 h per week, depending on the needs of the hospital. Based on various studies, the average working hours of nurses in Tehran and Qom provinces are 48.41 and 57.49, respectively[18, 19]. In studies in different countries, different findings have been reported about the labor supply of nurses. In Zhang et al.'s study among nurses working in ten hospitals in China (2021), the average daily working time was reported to be five hours for each nurse, while nurses tended to have an average work supply of four hours per day [20]. A study in Korea in 2019 mentioned the average work supply of nurses in three specialized hospitals as equal to 44.6 h per week [21]. In the study by Bae et al. [22] in hospitals with less than 300 beds in Korea, the results showed that nurses had an average work supply of 9.6 h per day, and according to working conditions, nurses working in intensive care units worked longer daily hours (equivalent to 10 h or more per shift). Reported labor supply for more than a quarter nurses working in public and private hospitals in the US was 12 h per day. A third of the total sample worked more than 40 h per week, and almost a quarter of nurses who had more than one job worked about 50 h per week [23]. The average working hours of nurses in the UK was 32.74 h per week [24]. In Thailand the lowest and the highest workload was 63 and 82 h per week respectively [25]. The reason for the difference in the quantity of nurse’s labor supply in different areas can be attributed to the difference in employment laws and regulations of countries or different methodology of estimating and calculating the labor supply.

The analytical findings showed that there was a negative relationship between nurses’ labor supply and their work experience. These results were in line with Kankaanranta and Rissanen’s study among Finnish nurses [17], Taei study on urban and rural women and men in Iran [26], Alshmemri in Saudi Arabia [27] and Antonazzo [8]. The reason for this can be mentioned the repetition of the work process, the reduction of economic and non-economic incentives, different tasks or roles that nurses acquire after years of service and more job burnout among people with higher experience.

Labor supply was positively related to marital status, so that single nurses had more labor supply than married ones. In other studies, such as Condliffe [9], Borjas [10], Rice in England [24], Askildsen in Sweden [28] and Kankaanranta and Rissanen [11] researches also the labor supply was higher in single people. Married people are often less willing to work overtime due to family-work conflicts, more busyness and responsibilities in their joint life. They also have less concentration and physical and mental strength for providing services to patients.

Satisfaction with the arrangement of work shifts had a negative relationship with the labor supply. It seems that nurses will be satisfied with the arrangement of work shifts if it reduces their working hours. The results of this study were contrary to the results of the studies of Ekici et al. [29] and Chan [4], in America and Askildsen [28], who stated that nurses who worked in variable shifts, reported higher levels of work-family conflict and workload than nurses who continuously worked day shifts. The study of Hanel et al. in Australia [30] and Costa in Italy [31] found the type of work shift to be one of the factors influencing the work supply of nurses and expressed the work supply in night shifts due to sleepiness and disruption is lower. The study of Letvak et al. in North Carolina showed that with incentive programs and policies and flexibility in choosing work shifts, labor supply by nurses can be increased [31].

The workload and pressure of the workplace also had a positive relationship with the labor supply among nurses; in such a way that the volume and pressure of work at the workplace was greater, nurses had to work more hours or shifts, which indicated the lack of nurses in the hospital. Also, our findings showed that satisfaction with the work environment increases the labor supply. In Jenkins et al.'s study, employees who were exposed to high work pressure provided significantly less work [32]. The results of the study by Ekici et al. in Turkey also showed that nurses who had high workload and pressure tended to leave their current job and work in organizations with better working conditions, less workload and more managerial support [29]. Seo in Korea [33] and Chan et al. in America [4] indicated that work pressure has a significant effect on nurses’ satisfaction and subsequently their labor supply. In fact, the work structure of nurses should be organized in order to increase their job satisfaction and supply more of their work.

The findings also showed that people with medium employment status (contractual employees) had significantly more and people with weak employment status (corporate employees) had significantly less labor supply than nurses with strong (formal) employment relationship. These results were in line with the study of Kankaanranta and Rissanen [17] and Rice [24]. This issue is due to the motivation to work more in people with average employment status to promote to the official employment status and the lack of motivation in nurses with poor employment status due to their unclear career future.

Spouse's income had a negative relationship with the labor supply of nurses. These results are in line with the studies of Simon [9], Kankaanranta and Rissanen in Finland [17], Borjas [10], Rice [24], Antonazzo [8], Brewer [34], Link [35], Link et al. in American Nurses [36] and Sloan [37]. In relation to this issue, it seems that nurses who have employed spouses with a better income status tend to reduce their labor supply due to less economic need.

In terms of the place of work, nurses in the emergency department had significantly less work supply. Tagrabi et al. (1400) in their study pointed out the significant relationship between the workplace and nurses’ job satisfaction [38]. In Kankaanranta and Rissanen’s study [11], nurses in outpatient departments, operating rooms or home care worked longer hours than nurses in other departments. It seems that due to the large number of patients, the lack of staffs and the high stresses in the environment of this department [39], some nurses working in these departments prefer to have less labor supply.

It is important to point out that our study was conducted after the Covid-19 pandemic, and this can affect the supply of nursing staff. Because the nurses were not satisfied due to high burnout, stress and high workload during the COVID-19 pandemic. However, this case was not investigated separately in our study and it can be considered as a suggestion for conducting another study in the future.

The most important limitation of the present study was the use of self-reported data. Especially regarding labor supply and income variables, there may not be complete accuracy in the participants' statements. In any case, this study is one of the few studies conducted in developing countries and Iran, which deals with the labor supply of nurses.

## Conclusion

In general, the labor supply of nurses working in public hospitals in Shiraz is affected by demographic, economic and non-economic factors, among which the most important factors were related to non-economic variables. It seems that the non-financial cost and benefits related to the job as well as internal factors have a more important effect on the labor supply of nurses. The work environment, organizational atmosphere, hospital communication and policies, nursing job status in the society, hospital facilities and equipment, non-financial benefits and even nurses’ job attitudes are among these. Therefore, it is recommended to consider these issues in order to increase labor supply and reduce the shortage of nurses, especially in developing countries.

## Data Availability

The datasets generated and/or analyzed during the current study are not publicly available due to data sharing policies of the vice chancellery for research affairs of Shiraz University of Medical Sciences but are available from the corresponding author on reasonable request.
